# Extracellular domain shedding of NOTCH3 during endocytosis associated with heterogeneity between different CADASIL mutant activation mechanisms

**DOI:** 10.1186/s12964-025-02362-1

**Published:** 2025-08-06

**Authors:** Samira Hosseini-Alghaderi, Martin Baron

**Affiliations:** School of Biological Sciences, University of Manchester, Michael Smith Building, Oxford Rd, Manchester, M13 9PY UK

## Abstract

**Background:**

Mutations in NOTCH3 cause CADASIL, a dominantly inherited condition, linked to recurrent stroke and vascular dementia and associated with accumulation of the ECD of NOTCH3. The latter has a toxic effect on VSMCs. Misregulated signalling may also play a role in disease progression. ECD detachment is an obligatory step in NOTCH3 activation, but some CADASIL mutants prevent ligand-induced activation and so ligand interactions are not a common underlying requirement. Here we investigated whether basal NOTCH3 endocytosis that is associated with ligand-independent activation mechanisms can be source of ECD shedding in CADASIL mutants.

**Methods:**

We used transient transfection of hTERT-RPE1 cells to express WT, R90C, C212Y and C455R mutant NOTCH3 constructs. Internalisation of NOTCH3 was followed using a pulse-chase endocytic uptake assay after surface NOTCH3 labelling of live cells. Immunolocalisation of NOTCH3 ECD and ICD was used to define the subcellular localisation of expressed NOTCH3 in the secretory and endocytic pathway of transfected cells, and endogenous NOTCH3 in MCF7 cells and VSMCs derived from human ES cells. To investigate NOTCH3 signalling we used a luciferase reporter assay under control of a NOTCH-responsive reporter element.

**Results:**

Both WT and CADASIL NOTCH3 proteins are endocytosed before ECD shedding and then undergo dissociation and independent trafficking of the ECD and ICD in the endosome. The relative amount of ICD compared to ECD that colocalised with endosomal markers increases as NOTCH3 progresses through the endosomal trafficking pathway from early endosome to lysosome. The R90C mutant showed earlier separation of ECD compared to WT or other CADASIL mutants tested. All WT and mutant constructs activated downstream signalling when expressed in hTERT-RPE1 cells, and these basal signalling levels were not affected by the C455R mutation which removes ligand-activated signalling. R90C showed distinctly different requirements for activation being less sensitive to metalloprotease inhibition and more sensitive to inhibition of the lysosomal protein TRPML.

**Conclusions:**

Basal NOTCH3 endocytosis and signalling is a potential source of ECD shedding and accumulation in CADASIL. Different mechanisms may apply to different CADASIL mutants and understanding the variety of mechanisms by which NOTCH3 signalling and ECD shedding occur will inform new targeted approaches to treatments of small vessel disease. Tuning NOTCH3 activity through modulation of the endocytic pathway may offer better tolerated approaches than direct targeting of NOTCH3 signalling.

**Supplementary Information:**

The online version contains supplementary material available at 10.1186/s12964-025-02362-1.

## Introduction

**Fig. 1:**
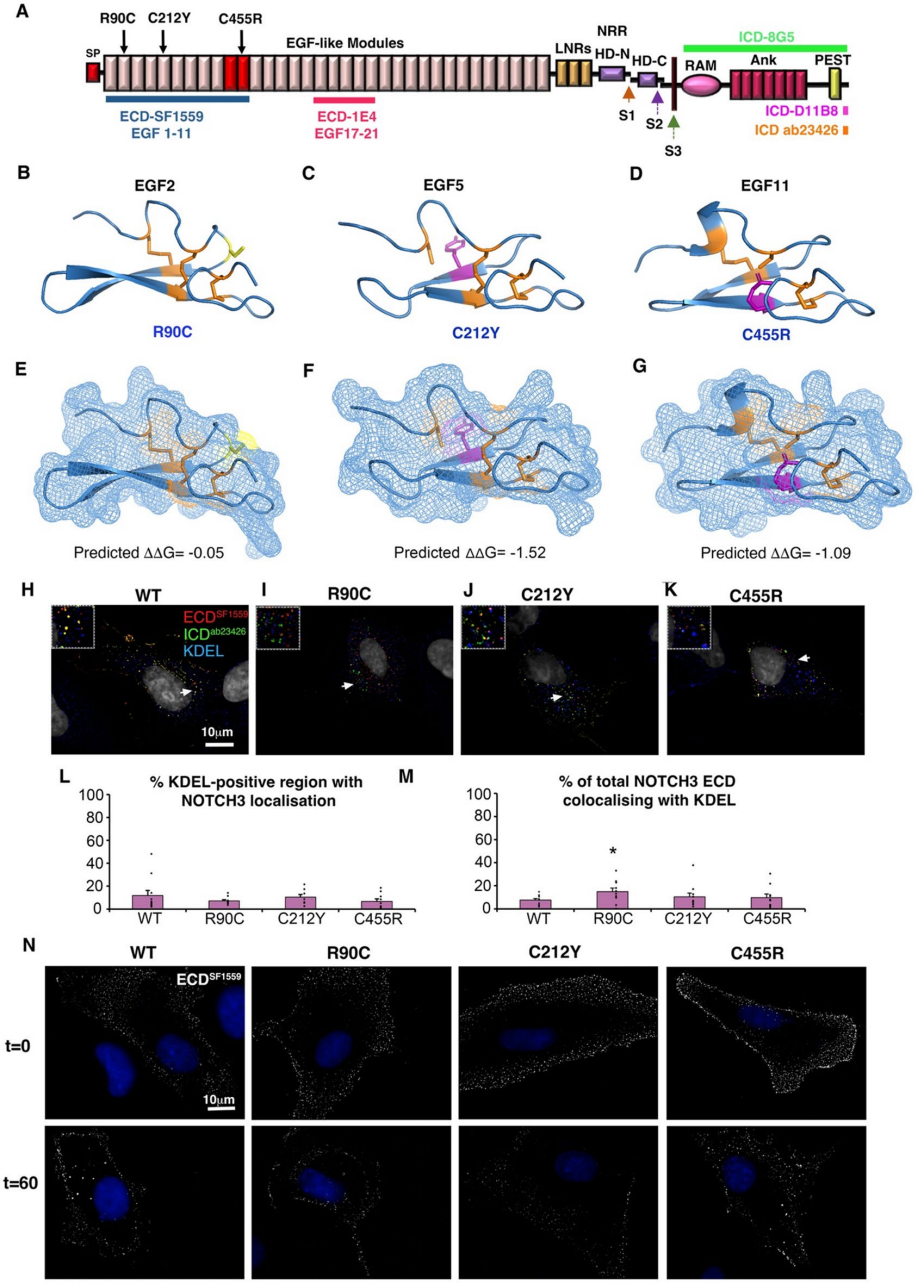
CADASIL NOTCH3 mutants are localised to cell surface like WT. **(A)** Modular structure of NOTCH3, SP indicates signal peptide, red shaded EGF-like modules indicate core ligand binding region, LNR indicates Lin12-Notch repeats, NRR is the negative regulatory region, S1, S2 and S3 are proteolytic cleavage sites, HD-N and HD-C are the heterodimer interface region. RAM and Ankyrin repeat region (Ank) are motifs involved in binding CSL transcription factors, PEST domain is involved in ubiquitin-dependent turnover of the ICD. Location of mutants used in this study are indicated by arrows. Coloured bars indicate locations of regions of NOTCH3 in which epitopes used in this study are located. **(B-D)** AlphaFold2 predictions of WT NOTCH3 EGF modules 2 **(B)**, 5 **(C)** and 11 **(D)**, and mutant substitutions introduced using the SDM programme. Location of the new Cys residue in R90C indicated in yellow, and other substitutions that replace cysteine residues are shown in magenta. **(E-G)** Surface views of the structures from (B-D) depicted as a surface mesh. The introduced cysteine in R90C is surface exposed **(E)**, but the free cysteines in C212Y and C455R are buried within the module structure (**F,G**). Negative ΔΔG values indicate predicted decreases in stability after mutant substitution [40]. **(H-K)** Expressed WT NOTCH3 **(H)** and CADASIL mutants **(I-K)** do not show strong accumulation in the ER when expressed in hTERT-RPE1 cells. **(L**,** M)** scoring of NOTCH3 localisation in KDEL-positive ER, by % area of ER occupied **(L)** and by % of total NOTCH3 staining intensity **(M)** in z-sections through the cytoplasmic region. R90C shows a small increase in %NOTCH3 which is localised to the ER. * indicates *P* < 0.05 by student t-test, error bars SEM, *n* = 10. **(N)** Antibody surface labelling of NOTCH3 localisation on non-permeabilised cells at time 0 and after 60 min chase during a live cell anti-ECD uptake assay. Similar time-zero surface distributions, and subsequent surface depletion of WT and CADASIL mutants can be seen

NOTCH3, one of four human Notch genes, is expressed in vascular smooth muscle cells (VSMCs) and contributes to VSMC development, maturation and phenotypic plasticity between their synthetic and contractile forms [1–7]. Certain missense mutations in NOTCH3 cause a dominantly inherited small vessel disease, CADASIL (Cerebral Autosomal Dominant Arteriopathy with Subcortical Infarcts and Leukoencephalopathy), which is associated with recurrent strokes and vascular dementia [8–10]. The prevalence of CADASIL is around 2–5 in 100,000 although recent genomics studies indicate a substantially higher proportion of the population have CADASIL-like NOTCH3 mutations and hence milder forms of inherited small vessel disease may be underdiagnosed [11]. CADASIL is a progressive disorder with an onset during middle age and is characterised by accumulation of the NOTCH3 Extracellular domain (ECD) [12], which comprises of 34 tandem Epidermal Growth Factor (EGF)-modules (Fig. 1A). EGF modules are defined by three conserved disulphide bonds [13] and archetypal CADASIL mutations add to or remove a cysteine residue in one of these EGF repeats [8]. There is heterogeneity of disease outcomes reported depending on the EGF-module affected, with EGF 1–6 mutations normally resulting in more severe and earlier-onset disease than mutations in other regions of the ECD [14]. CADASIL is associated with degeneration of vascular smooth muscle cells (VSMCs) but the underlying links to NOTCH3 misregulation are unclear and may be multifactorial. A combination of altered NOTCH3 signalling [15–17] and toxicity resulting from accumulation of Granular Osmiophilic Material (GOM) deposits, which include NOTCH3 ECD and other associated proteins [18, 19], may contribute to disease progression.

NOTCH3 is an attractive target for developing therapies linked to VSMC misregulation. Indeed, there is already significant interest in targeting Notch proteins for other therapeutic applications, including cancer [20]. However, direct targeting of the Notch signal to switch off the pathway can have severe side effects [21]. The level of risk that is associated with such treatments is not appropriate for chronic, progressive diseases that are not immediately life threatening, like CADASIL, making finding effective treatments problematic. Understanding how to manipulate NOTCH3 signalling in more subtle ways can provide novel routes towards new therapies. ECD-detachment is a normal part of the Notch-signal activation process and it is likely that NOTCH3 activation mechanisms are a source of detached ECD that accumulate in CADASIL. As with other Notch family proteins, NOTCH3 signalling is initiated by ligand binding, which results in proteolytic removal of the ECD by the metalloprotease ADAM10, and subsequently the release of intracellular domain (ICD) from the membrane by gamma-secretase [7]. The released ICD relocates to the nucleus to bind to and activate the CSL (CBF, Su(H), Lag2) transcription factor. Mutations that do or do not disrupt ligand binding and ligand-induced signal induction have been shown to cause CADASIL, indicating that altered ligand-dependent interactions are not a common underlying factor [22–24]. In *Drosophila melanogaster*, ligand-independent Notch activation is associated with endocytosis of full-length Notch and ectodomain shedding of the ECD within the endocytic pathway, as a precursor to gamma-secretase release of the intracellular domain from the endosomal membrane [25]. Work on *Drosophila* Notch has shown that the endocytic pathway has the capacity to tune the level of Notch activity, both up and down, through modulating surface exposure of Notch to ligands, and regulating the activity of ligand-independent activation of Notch localised on the endosomal surface, to provide robustness to environmental and genetic perturbation [25, 26]. NOTCH3, has also been demonstrated to undergo ligand-independent activation although the mechanism has not been explored [27, 28]. We therefore investigated the endocytic trafficking and processing of NOTCH3 and the consequences on these processes of differently located CADASIL mutants. We found that both wildtype (WT) and CADASIL mutant NOTCH3 proteins traffic normally to the cell surface and display a punctate distribution on the cell membrane, from where NOTCH3 is endocytosed into the cell. In both WT and CADASIL mutants we found that the ICD becomes separated from the ECD during endocytosis and ICD subsequently localises independently of the ECD to the late endosome/lysosome. Expressed WT NOTCH3 undergoes basal activation, independently of ligands but dependent on metalloprotease activity. Inhibition of the latter activity reduced ECD separation resulting in more full-length NOTCH3 in the late endosome. CADASIL mutants R90C, C212Y and C455R are also activated by this basal NOTCH3 activity. However, we found heterogeneity of activation mechanisms depending on the mutant tested. The R90C mutant switched its activation mechanism to a metalloprotease-independent and TRPML-dependent mechanism, while C212Y and C455R activated by a metalloprotease-dependent mechanism like WT. This altered R90C mechanism was associated with differences in immunofluorescence localisation that suggest the ECD is detached before reaching the EEA1-positive endosome, unlike WT and the other CADASIL mutants tested. Different endocytic ectodomain shedding mechanisms and signal activation pathways may be relevant as a source of the long-term accumulation of the mutant NOTCH3 ECD in VSMCs and for understanding heterogenous pathological outcomes in CADASIL patients.

## Materials and methods

### DNA plasmid constructs

NOTCH3 expression vectors were pcDNA3.1-Notch (WT, R90C, C455R, and C212Y), a kind gift from Tao Wang (University of Manchester, UK). For empty vector control pcDNA3.1 was used (Thermo Fisher Scientific, Waltham, MA, USA). FP1 and FP2-NOTCH3 expression vectors were constructed in RP-HygroCMV (VectorBuilder Inc, IL, USA). FP1-NOTCH3 has in frame inserts of mTagBFP2 and mEGFP after amino acid 1377 and 2129, respectively. FP2-NOTCH3 has in frame insertions of mScarlet-I and mNeonGreen at the same locations. For luciferase assays CBF-luciferase Notch reporter plasmid, was a kind gift from Keith Brennan (University of Manchester, UK) [29], for constitutive Renilla expression we used pRL-CMV (Promega, Wi, USA).

### Cell culture

hTERT Human Retinal Pigmented Epithelium-1 (hTERT-RPE-1, Clontech, CA, USA) cells were maintained in Dulbecco’s Modified Eagles Medium combined with high concentration of glucose, amino acids and vitamins with F-12 (DMEM, Sigma-Aldrich, MO, USA) supplemented with 1% penicillin-streptomycin (Sigma-Aldrich, MO, USA), 1% L- glutamine (Gibco, MA, USA) and 5% felt bovine serum (Sigma-Aldrich, MO, USA). Cells were fed every 2/3 days with fresh medium and incubated at 37 °C in a humidified atmosphere containing 5% CO2. Experiments were conducted using cells at passage 6–18. hTERT-RPE-1 cells were obtained from Philip Woodman (University of Manchester, UK).

Michigan Cancer Foundation-7 (MCF-7) cells, (HTB-22, ATCC, VA, USA) were maintained in DMEM with low glucose and L-Glutamine supplemented with 1% penicillin-streptomycin (Sigma-Aldrich, MO, USA) and 5% fetal bovine serum (Sigma-Aldrich, MO, USA) in T75 culture flasks (Corning, NY, USA). Cells were fed every 2/3 days with fresh medium and incubated at 37 °C in a humidified atmosphere containing 5% CO2. Experiments were conducted using passage 16–20 MCF-7 cells.

Human embryonic stem cell (hESC) line used was (Man-13) [30]. hESCs maintained in mTeSR^TM^1 Basal Medium (#85851 STEMCELL Technologies, BC, Canada) supplemented with mTeSR^TM^1, 5X Supplement #85,852 (STEMCELL Technologies, BC, Canada). Cells cultured on six-well plate (Corning, NY, USA) coated with 2 ml supplemented mTeSR^TM^ 1 and 10ng Vitronectin (rhVTN-N, A14700, Life Technologies, Paisley, UK) and 10µM Rock Inhibitor (Y-27632, STEMCELL Technologies, BC, Canada) per well of a six-well plate (Corning, NY, USA). Cells were fed using fresh media every day.

Man-13 cells were differentiated into VSMCs using an 18-day differentiation protocol. 6–8 clusters/cm^2^ of Man-13 cells were seeded onto a VTN-N, 6-well plate in E8 (Gibco, MA, USA) medium supplemented with Y-27,632 ROCK Inhibitor (10µM) for 24 h. Man-13 cell culture media was then swapped to E6 (Sigma-Aldrich, MO, USA) supplemented with 10µM SB-431,542 (Tocris, Bioscience, Abingdon, UK) and 10ng/ml FGF2 (Peprotech, NJ, USA). The medium was replaced every 24 h with the same supplement for 5 days. On day 5, cells were washed with PBS without Ca^2+/^Mg^2+^ and dissociated by 1 ml 0.5% EDTA/PBS. Cells were centrifuged at 500 rpm for 5 min and then cell pellet was resuspended in E6 medium and seeded onto VTN-N coated six-well plates at 0.5 × 10^4^ cells. E6 medium was supplemented with 2ng/ml TGF-ß1 (Peprotech, NJ, USA) and 5ng/ml PDGF-ß (100-14B, Peprotech, NJ, USA). The medium was replaced every 24 h with the same supplement until day 18 of differentiation.

### Cell transfection

Prior to transfection, cells were cultured and seeded on 0.01% poly L-lysine-coated coverslips in a twelve-well or six-well plates (Corning, NY, USA) as required for 24 h Cells at 37 °C to reach 50% confluency. Cells were transfected with desired plasmid and Genejuice^®^ Transfection Reagent (Merck Millipore, MA, USA) at 1:3 ratio. First, 3 µl of Genejuice^®^ transfection reagents were diluted in 100ul Opti-MEM (Thermo Fisher Scientific, MA, USA) per sample and incubated at room temperature for 5 min. In the next step, the desired amount of DNA was added to the dilution and incubated at room temperature for 15–20 s to form a transfection complex. Then cells were washed with Opti-MEM media and exposed to transfection complex with an additional 900ul Opti-MEM media per well in six-well plates (Corning, NY, USA), for 2 h, and then media was changed into fresh culture media.

### Antibodies

Primary antibodies used were polyclonal sheep anti-NOTCH3 ECD (SF1559, R&D systems, Minneapolis, MN, USA, used 1:500), monoclonal mouse anti-NOTCH3 ECD (1E4, Merck Life Science, Gillingham, UK, used 1:500), monoclonal Rabbit anti-ICD (D11B8, Cell Signaling technology Inc, MA, USA, used 1:500), polyclonal Rabbit anti-ICD (ab23456, Abcam, Cambridge, UK, used 1:500), monoclonal rat anti-ICD (8G5, Cell Signaling technology Inc, MA, USA, used 1:500), monoclonal mouse anti-EEA1 (E9Q6G, Merck-Millipore, MA, USA, used 1:200), monoclonal mouse anti-CD63 (RFAC4, Merck Life Science, Gillingham, UK, used 1:500), monoclonal rabbit anti-LAMP1 (D2D11, Cell Signaling technology Inc, MA, USA, used 1:500), monoclonal mouse anti-KDEL (10C3, ENZO Life Sciences, NY, USA, used 1:500), polyclonal anti-Rab11a (2413 S, Cell Signaling Technology Inc, MA, USA, used 1:50), mouse monoclonal anti α-smooth muscle actin (1A4, Abcam, Cambridge, UK, used 1:500), polyclonal rabbit anti-Calponin, CNN1 (ab46794, Abcam, Cambridge, UK, used 1:500), monoclonal anti-Oct4 (9B7, R&D systems, MN, USA, used 1:500), mouse monoclonal anti-SOX2 (245610, R&D systems, MN, USA, used 1:500). Fluorescent-conjugated secondary antibodies were obtained from Invitrogen (CA, USA, used 1:500).

### Immunofluorescent (IF) staining

Cells were washed with 2 ml PBS prior to fixation with 1 ml 4% paraformaldehyde (PFA) (PierceTM, Cat. no. 28906, Thermo Fisher Scientific,) in PBS for 20 min at room temperature, and then incubated with 0.25% NH_4_Cl (Sigma Aldrich, MO, USA) for blocking PFA residues. Cells were washed again with PBS and then, to permeabilise, cells were incubated with 1 ml 0.1% Triton X-100 (T9284, Sigma-Aldrich, MO, USA) in PBS for 10 min following 2x wash with PBS. Next cells were incubated with 1 ml 5% NDS (normal donkey serum, Jackson ImmunoResearch, PA, USA) in PBS for 30 min to block any non-specific binding. Cells were incubated with 500 µl of diluted primary antibody in blocking solution for 1 h at room temperature and then washed three times with 1 ml solution and then incubated with 500 µl of diluted secondary antibody in solution for 45 min at room temperature.

### Antibody uptake assay

To label surface NOTCH3, the transfected cells were washed with 1 ml of chilled fresh culture media on ice and then incubated with anti-ECD antibodies for 15 min on ice to slow down NOTCH3 endocytosis. Then cells were washed with chilled culture media followed by 1 ml of warm fresh culture medium. Wells were then incubated at 37^o^C/5%CO2 for various chase times. Coverslips were collected at each time point, washed with 1 ml 1x PBS at room temperature and fixed with 4%PFA in PBS for 20 min at room temperature. Cells were then blocked with 5%NDS/PBS for 30 min and subsequently incubated with desired secondary antibodies (1:500) for 45 min in dark at room temperature. Cells were then rinsed with 1 ml of 5%NDS/PBS twice followed by one wash with 1 ml PBS. Stained cells were then mounted in Vectorshield with DAPI (Vector Laboratories, CA, USA) and left at 4 °C overnight. To label endocytosed NOTCH3, after the chase period, cells were fixed/permeabilised, as described above, and treated with anti-NOTCH3 ICD and endosomal pathway markers (anti-CD63 or anti-EEA1) followed by secondary antibodies.

### Immunofluorescence imaging

Images were captured using Volocity (Perkin Elmer, Beaconsfield, UK) with an Orca-ER digital camera (Hamamatsu Photonics, Hammamatsu city, Japan) mounted on a M2 fluorescent microscope (Carl Zeiss, Oberkochen, Germany). Deconvolution was performed with three nearest neighbours using Openlab (Improvision/Perkin Elmer, Beaconsfield, UK) and processed in Photoshop (Adobe, CA, USA). For quantitation of colocalization in hTERT-RPE1 cells, Z-section images were recorded at x100 and deconvolved using Openlab with 3 nearest neighbours to remove out of focus signal. An image plane was selected midway through the cell body at the plane of focus for the nucleus. Images were uploaded to ImageJ using FIJI [31, 32] and colocalization quantified as Pearson’s coefficients using the JACOP (Just another colocalisation Protocol) plug-in [33]. For quantification of colocalisation in cytoplasmic organelles in MCF7 and VSMC cells, a mask was created around the perimeter of the nucleus to exclude nuclear-localised anti-ICD staining from the quantification. For quantification of NOTCH3 accumulation in the ER, ImageJ was used to calculate the % area of KDEL staining occupied by NOTCH3, and the % of total intensity of NOTCH3 staining which was coincident with KDEL staining regions. For calculation of ECD and ICD separation in the early endosomes, EEA1-positive organelles were scored for number of puncta present as full-length, ECD-only and ICD-only localisations.

### Luciferase Notch reporter assay

The Dual-Luciferase^®^ Reporter (DLRTM, E1910, Promega, WI, USA) Assay System was used to measure NOTCH3 signalling activity. pRL-CMV is a Renilla luciferase-expression (Promega, WI, USA) vector driven by a CMV early promoter to provide constitutive transcription to act as an internal control for cell transfection efficiency and cell viability. p10xRBPJ-luc is a Firefly luciferase reporter vector which has 10 copies of the canonical CBF/RBPJ transcription factor binding sites upstream of the firefly luciferase cDNA and acts as a Notch Response Element (NRE) [29]. Both reporter vectors were co-transfected with the NOTCH3 plasmid in cell culture to generate stabilised luminescence signals for both vectors in the same samples. The ratio of the two signals provides relative quantification of NOTCH3 activity.

### Use of inhibitors

For treatment with inhibitors, MCF7 or hTERT-RPE1 cells were cultured for 48 h with 10µM inhibitor in 2 ml culture medium, which was replaced after the first 24 h. Inhibitors used were DAPT (Cell Guidance Systems, MO, USA), ML-SI1 (Scientific Laboratory Supplies, Nottingham, UK), BB94 (Stratech Scientific, Ely, UK), RO4929097 (Selleckehem, TX, USA).

## Results

### Expressed CADASIL mutants are trafficked to the cell surface similar to WT

We studied the consequences on NOTCH3 of three CADASIL mutations, R90C, C212Y and C455R. R90C and C212Y mutations are located within the N-terminal EGF1-6 region (Fig. 1A) where the majority of CADASIL mutations are located [8, 34, 35]. C455R was chosen as it is a mutation in the ligand-binding region of NOTCH3 (Fig. 1A) which strongly inhibits ligand-dependent NOTCH3 activation [22, 24]. The R90C mutation is in the top six of the most frequently occurring CADASIL mutants [36] and C212Y is notable for spinal cord lesions in addition to typical CADASIL features [34]. The structural consequence of CADASIL mutations on the affected EGF domains of NOTCH3 have not been characterised in detail. It is possible that that such mutations disrupt the EGF-fold and this leads to reduced delivery of NOTCH3 to the cell surface [37]. We used the structure prediction programme AlphaFold2 [38, 39] to predict the structures of three wild type EGF modules from NOTCH3 in which the CADASIL mutants chosen are located, EGF2, EGF5 and EGF11. We then used the Site Directed Mutator (SDM) programme [40] to predict the consequences of R90C, C212Y, and C455R (Fig. 1B-D). While a small decrease in stability was predicted for the three mutations (Fig. 1E-G), the results showed that the missense mutations were accommodated with relatively little perturbation to the structure. Exposure of an unpaired cysteine residue on the surface of the EGF-module structure was only evident for the new cysteine introduced in the R90C substitution (Fig. 1E-G).

When NOTCH3 was expressed in hTERT-RPE1 cells, previously used for similar studies of protein trafficking [41], we did not observe strong accumulation of CADASIL mutant NOTCH3 in the endoplasmic reticulum (ER), compared to WT, that would indicate significant protein misfolding in the secretory pathway (Fig. 1H-K). When we quantified ECD localisation we found no significant difference in the proportion of KDEL (K-Lysine, D-Aspartic acid, E-Glutamic acid, L-Leucine)-positive ER that was occupied by NOTCH3 immunolocalisation, although there was a small increase in the amount of R90C localised to ER measured by fluorescence intensity (Fig. 1L, M). Surface staining of NOTCH3 localisation on live, non-permeabilised cells with an anti-ECD antibody showed a similar punctate surface distribution of WT and CADASIL mutant NOTCH3, which was depleted from the cell-surface over a one-hour chase period (Fig. 1N).

### ECD separation after endocytosis of full-length NOTCH3

**Fig. 2:**
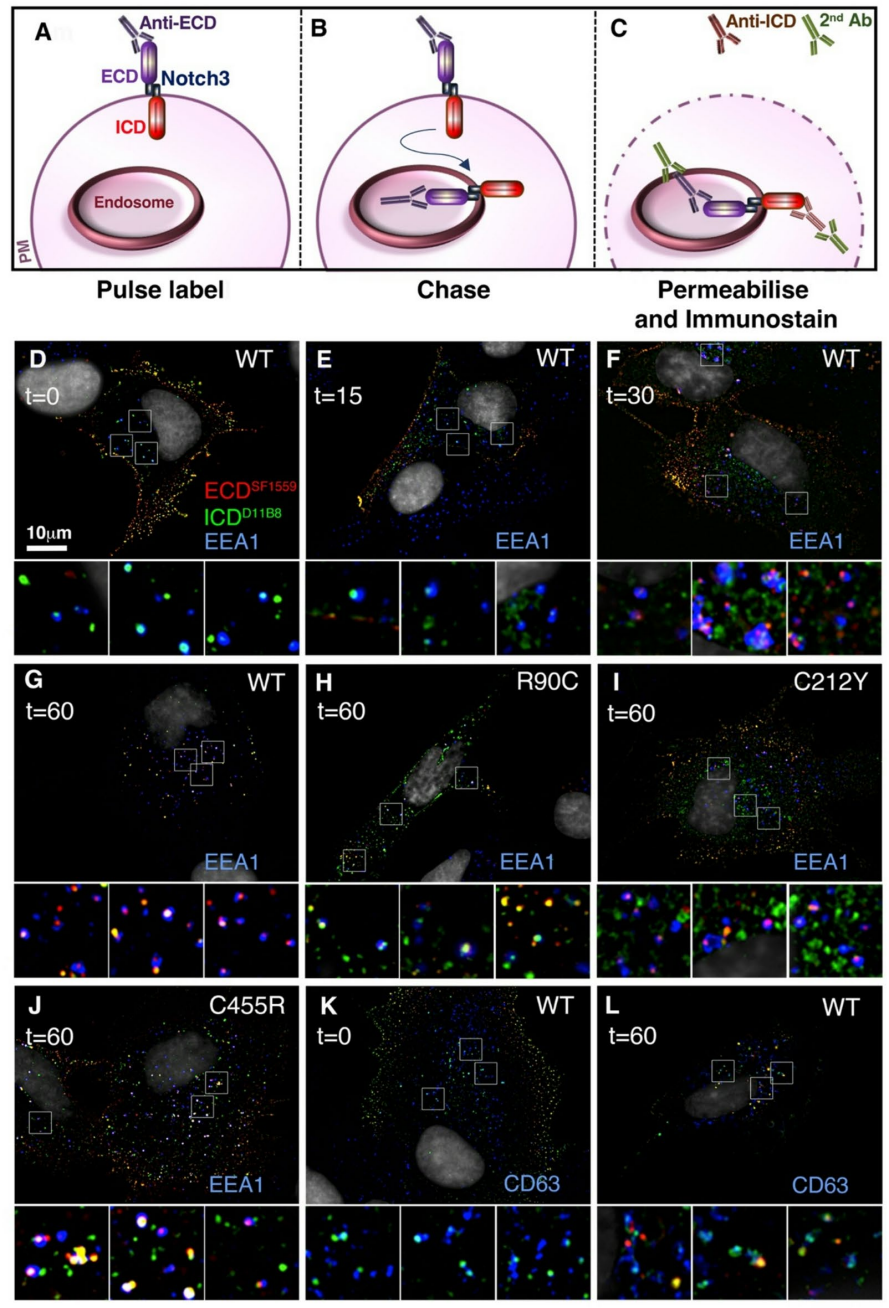
Full-length WT and CADASIL mutant NOTCH3 are endocytosed similarly. **(A-C)** Schematic view of pulse-chase labelling protocol. **(D-G)** WT NOTCH3 localisation in permeabilised cells after antibody uptake at 0 **(D)**, 15 **(E)**, 30 **(F)**, and 60 **(G)** minutes. Little surface-labelled NOTCH3 is transported to the early endosome by 15 min chase but is evident in EEA1 positive endosomes by 30 and 60 min. Presence of both full-length and ECD-only staining suggests ECD-shedding can occur by the time NOTCH3 is localised to the EEA1-marked endosome. **(H-J)** CADASIL mutant NOTCH3 localisation after 60-minute chase shows the mutant proteins labelled at the cell surface also become localised to the EEA1-positive endosome. **(K**,** L)** After 60-minute chase surface, little labelled NOTCH3 ECD has reached the CD63-positive late endosome while ICD is present. However full-length NOTCH3 and ECD-only localisations are found adjacent to CD63-marked organelles

To investigate the fate of internalised NOTCH3, the uptake experiment was repeated and cells were further permeabilised and immunostained with anti-ICD and appropriate secondary antibodies after different chase intervals (Fig. 2A-C). Up to the 15-minute chase time point we only observed ICD localised in EEA1 (Early Endosome Antigen 1)-positive early endosomes, consistent with lack of access of the anti-ECD to intracellular compartments during the pulse-labelling period (Fig. 2D, E). Notch ICD was found to be localised in puncta on the EEA1-positive organelle. Notch ECD staining appeared in the organelle at 30 and 60-minutes time points, either colocalised with ICD as full-length Notch, or was present without ICD staining (Fig. 2F, G), the latter suggesting separation of ECD from ICD had occurred. As it has previously been shown that mammalian Notch proteins, unlike *Drosophila* Notch, progress to the cell surface as a heterodimer following Furin-dependent cleavage in the Golgi [42–44] then we refer to the S1 processed heterodimeric form throughout this work as “full-length”. The ECD of the three CADASIL mutants was also found localised within the early endosome after endocytic uptake and 60 min chase (Fig. 2H-J). Therefore, these mutants follow a similar endocytic uptake to WT NOTCH3, even when the ligand-binding site is perturbed by the C455R mutant. The pulse chase experiment was repeated with a one-hour chase to investigate the localisation of WT NOTCH3 within CD63-positive late endosomes (Fig. 2K, L). After a 1 h chase period, late endosomes were predominantly stained with anti-ICD, again in punctate subdomains within the CD63-positive structures (Fig. 2L). There was little ECD colocalised with CD63, but outside of these late endosome structures both full-length NOTCH3 and separate ECD and ICD localisations were observed (Fig. 2L).

These results indicate that surface-localised WT and CADASIL NOTCH3 is endocytosed as a full-length protein. The presence of ECD-only stained puncta within the early endosome suggests that this may be a location where ECD shedding occurs. An alternative explanation could be that ECD is detached at the cell surface through ligand interaction and trans endocytosed into adjacent cells. However, the limited transfection frequency of the hTERT-RPE-1 cells meant that transfected and non-transfected cells were often adjacent to each other and in these cases we did not observe ECD transferred into the adjacent non-transfected cell (for example, Fig. 2E and G). In addition, where cells were at low density and not in contact with neighbour then internalisation of NOTCH3 ECD was still observed (for example, Fig. 2I and L). The C455R mutant, which disrupts ligand activation of NOTCH3, also internalised similarly to wild type. We could not, however, distinguish whether ICD-only puncta in the early and late endosomes represented full-length NOTCH3 which was inaccessible to anti-ECD during the antibody-labelling period, or were products of separation of ECD from ICD.

**Fig. 3:**
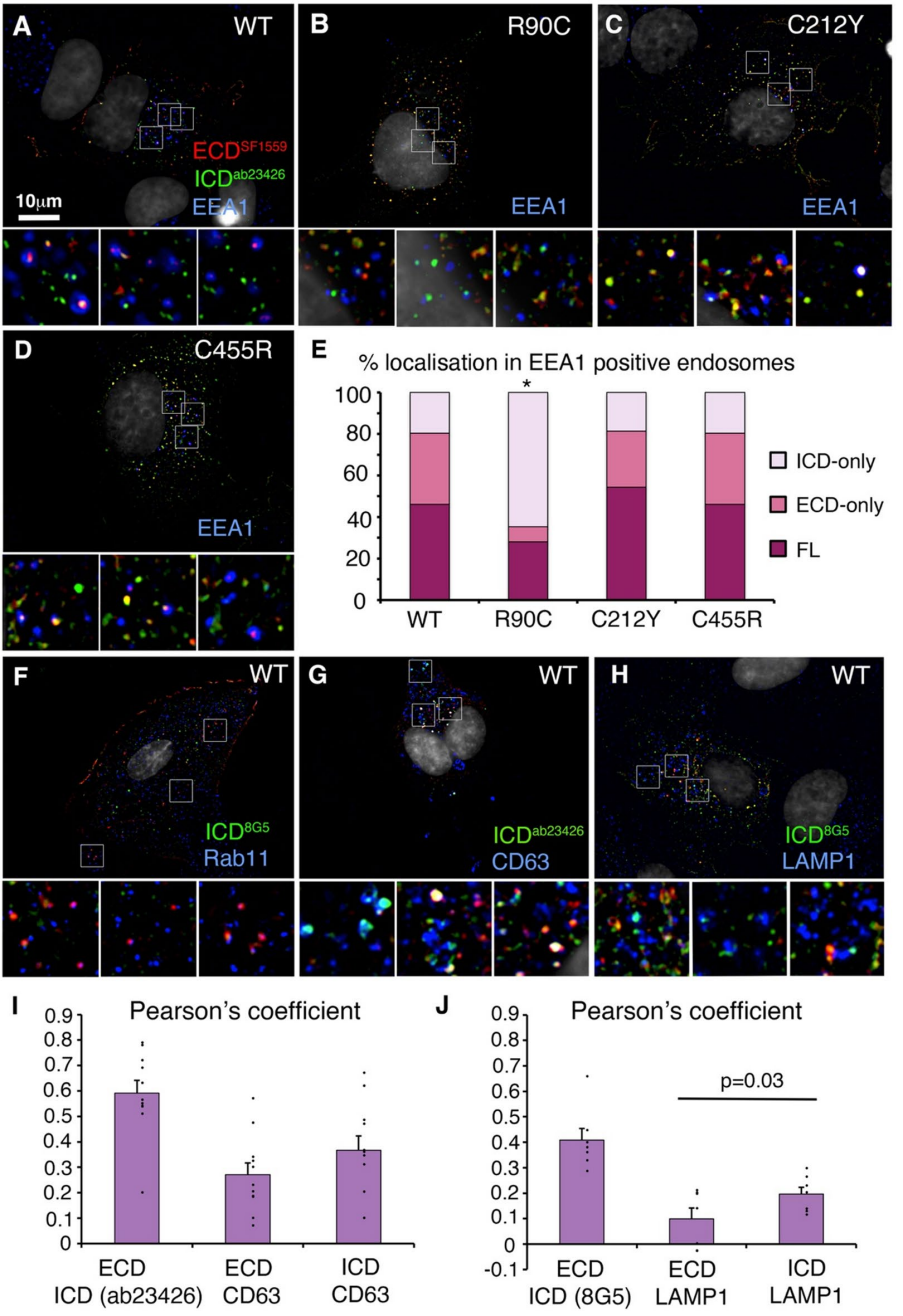
ECD-shedding in the early endocytic pathway. **(A-D)** Immunolocalisation of NOTCH3 with anti-ECD and anti-ICD compared with EEA1-marked endosomes in fixed and permeabilised cells of WT **(A)** and CADASIL mutants (**B-D)**. **(E)** Quantitation of NOTCH3 localisation in the early endosome, showing % endosomal-located NOTCH3 puncta which are either full-length NOTCH3, ECD-only, or ICD-only staining. R90C is distinguished from WT and other mutants by a greater proportion of ICD-only puncta compared to ECD or full-length. For WT, *n* = 91 NOTCH3 puncta from 198 endosomes scored. For R90C, *n* = 139 NOTCH3 puncta from 180 endosomes scored. For C212Y, *n* = 140 NOTCH3 puncta from 240 endosomes scored. For C455R, *n* = 164 NOTCH3 puncta from 302 endosomes scored. Data obtained from z sections from a minimum of 7 cells. * indicates *P* < 0.05 by chi squared test compared to WT. **(F)** Full-length NOTCH3, or ECD-only puncta located within or adjacent to Rab11-positive endosomes. **(G**,** H)** In CD63-positive endosomes **(G)** and LAMP1-positive lysosomes **(H)**, NOTCH3 localisation is predominantly as ICD-only puncta, quantified in **(I**,** J)**. Error bars in I and J are SEM, *n* = 11 and *n* = 7 respectively. Indicated statistical significance by student t-test

To investigate further the endosomal localisation of NOTCH3, we immunostained permeabilised and fixed cells with anti-ECD and ICD, along with compartment markers for early endosome (EEA1), late endosome (CD63), recycling endosome (Rab11) and lysosome (LAMP1) (Fig. 3A-H). In the EEA1-positive early endosome, WT NOTCH3 was predominantly either full-length or ECD-only localisation (Fig. 3A). Full-length or ECD-only staining was also located within, or adjacent to, Rab11 marked recycling endosomes (Fig. 3F). In contrast, in the CD63-labelled compartments we found both full-length and ICD-only spots (Fig. 3G, I). Similarly, in LAMP1 marked lysosomes we predominantly found anti-ICD staining (Fig. 3H, J).

Interestingly, we found differences in the processing and localisation of the different CADASIL mutants in the early endosome. While C455R and C212Y mutants showed similar localisation of full-length and ECD-only puncta of NOTCH3 in the early endosome (Fig. 3C-E), R90C showed a significantly higher proportion of ICD-only puncta and a decreased proportion of ECD-only spots (Fig. 3B, E). Localisation of the NOTCH3 CADASIL mutants in the late endosome and lysosome was predominantly ICD-only immunostaining like WT (Supplementary Fig. S1 compared with Fig. 3G, H). Interestingly western blots of cell lysates from NOTCH3 expressing cells also showed a distinctive protein band pattern for R90C which displayed two additional higher molecular weight bands not observed in WT, C212Y and C455R mutants (Supplementary Fig. S2A). A similar upshift of the R90C band was observed when expressed in hTERT-RPE1 cells (Supplementary Fig. S2B).

These results indicate that full-length NOTCH3 undergoes endocytic uptake and subsequent ECD removal, and that these processes are not affected by perturbation of the ligand binding site by the C455R mutation. There is a heterogeneity of outcomes arising from different CADASIL mutants, with the R90C mutation altering the location in which ICD localises independently from ECD. A common feature between WT and all three CADASIL mutants is the localisation of ICD staining in a punctate distribution in the late endosome and lysosome. This likely represents membrane anchored ICD after ECD removal, since gamma-secretase S3 cleavage would release soluble ICD for traffic to the nucleus where it has short half-life.

**Fig. 4:**
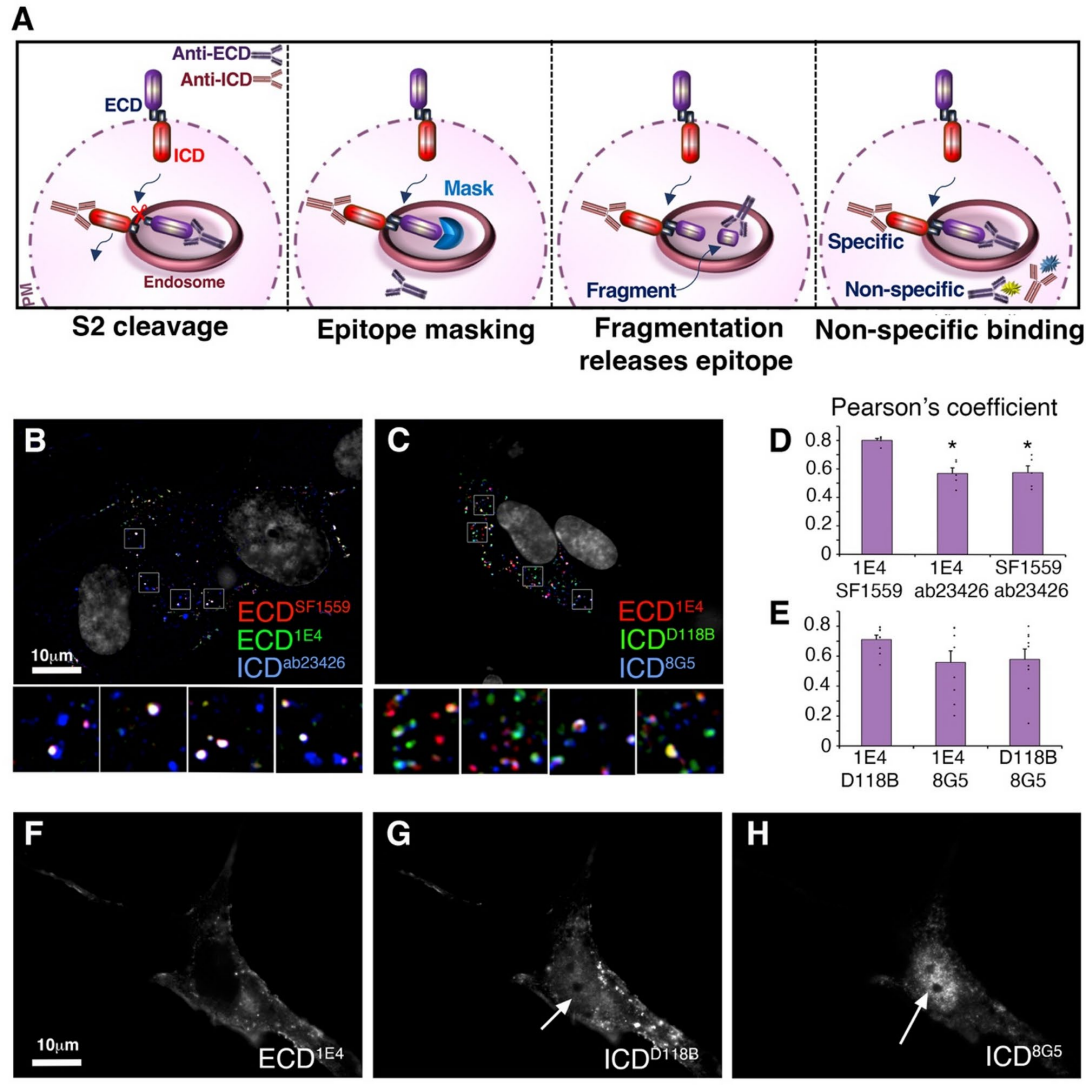
Visualisation of ECD/ICD independent localisation is not due to epitope masking. **(A)** Schematic diagram illustrating alternative explanations for visualisation of separated ECD and ICD-positive puncta, including: ECD shedding by S2 cleavage; epitope masking; fragmentation to remove terminal epitopes; non-specific staining. **(B)** Colocalisation of two different ECD epitopes compared to ICD^ab23426^. ICD-stained puncta can be observed which lack both ECD epitopes. **(C)** Colocalisation of two different ICD epitopes compared to anti-ECD^1E4^. ECD-only spots are present which lack both ICD epitopes. **(D**,** E)** Quantification of the colocalisation between the epitopes used in B, C. In D * indicates *P* < 0.05 compared with double ECD epitope colocalisation by student t-test. Error bars represent SEM, *n* = 5 **(D)**, *n* = 14 **(E)**. **(F-H)** Both ICD epitopes are located to the nucleus in NOTCH3 transfected cells but not the ECD epitope

We considered whether differential epitope accessibility to anti-ECD and ICD antibodies might be a reason for observing separated ECD and ICD localisations. This may arise by epitope masking, partial fragmentation to release an epitope, or non-specific staining of either or both antibodies (Fig. 4A). To address this, we conducted immunostaining with two different ECD antibodies raised against different regions of the protein with a single ICD antibody and vice versa. We found that when two ECD antibodies were used (SF1559 and 1E4) then ICD-only (ab23426) spots were still detected, lacking staining by both ECD antibodies (Fig. 4B, D). This indicates that epitope masking of the ECD is unlikely to account for observation of separate ICD staining. Furthermore, the colocalisation between the two ECD epitopes was significantly greater than the colocalisation between either ECD epitope and ICD, as would be expected if the ECD and ICD were separating. These data therefore rule out significant epitope masking or proteolytic removal of just the N-terminal epitope. When we stained NOTCH3 expressing cells with two different ICD antibodies (D11B8 and 8G5) and a single ECD antibody (1E4), then ECD-only stained spots were still present lacking both ICD epitopes (Fig. 4C, E), indicating that ICD epitope masking was unlikely to account for observation of separated ECD. Both ICD epitopes, but not the ECD epitope, were also detectable in the nucleus of NOTCH3 transfected cells (Fig. 4F-H). However, the colocalisation of the two ICD antibodies was not greater than the colocalisation of either epitope with the ECD and we saw less colocalisation of the 8G5 epitope with the ECD than D11B8 with the ECD (Fig. 4E). This suggested that epitope masking of the 8G5 epitope was occurring. To investigate this further we examined 8G5 localisation in the early endosome. Interestingly we found that unlike D11B8, 8G5 was rarely detectable in the early endosome or at the cell membrane (Supplementary Fig. S3), while it was clearly present in the lysosome (Fig. 3H, Supplementary Fig. S1D-F). Epitope masking therefore seems likely for 8G5 epitope in the early endosomal trafficking pathway. However, we were able to rule this explanation out for D11B8 and ab23426 epitopes. All the antibodies stained NOTCH3 transfected cells at a level well above any background staining in non-transfected cells, ruling out off-target immunostainings as an explanation for apparent epitope separation of expressed NOTCH3.

We also utilised fluorescent protein tagged NOTCH3 constructs (Supplementary Fig. S4A, B) to provide an alternative means to label the ECD and ICD, independent of antibody staining. FP1-NOTCH3 had mGFP-tagged ICD and mTagBFP2 located in the ECD (not visible in fixed samples). Non-permeabilised cells were stained with anti-NOTCH3 ECD. Colocalisation of anti-ECD immunostaining and GFP fluorescence confirmed that NOTCH3 located at the plasma membrane was full-length protein (Supplementary Fig. S4C). In the FP2-NOTCH3 construct, the FP tags were mScarlet-I for the ECD and mNeon-Green for the ICD, which were chosen for their fast folding and pH resistance properties [45, 46]. Consistent with the results using immunostaining, the FP2-tagged NOTCH3 showed separation of the ECD and ICD when transfected into hTERT-RPE1 cells (Supplementary Fig. S1D). Furthermore, after costaining of permeabilised cells expressing FP2-NOTCH3 with anti-ECD, mNeon-Green labelled ICD puncta could be observed that lacked both anti-ECD staining and mScarlet-I fluorescence (Supplementary Fig. S4D). The ECD antibody and mScarlet-I fluorescence showed a good colocalisation in cytoplasmic puncta, but at the outer cell membrane FP-tag fluorescence was weak compared to antibody staining. Nevertheless, regions of anti-ECD and mScarlet-I co-staining could also be observed at the cell membrane along with mNeonGreen fluorescence (Supplementary Fig. S4D). The combined results using FP-NOTCH3 and anti-Notch antibody labelling are therefore consistent with ECD shedding within the endosomal trafficking pathway.

### Endogenous NOTCH3 undergoes ECD separation in the endosomal pathway

We next investigated whether the observed ECD/ICD separation in the endosomal pathway was a feature that resulted from elevated levels of NOTCH3 expression after transfection. For this we used two cell types with endogenous NOTCH3, the human breast tumour cell line MCF7 and human VSMCs derived by differentiation of hESCs. To test the specificity of anti-NOTCH3 antibodies for immunostaining at endogenous expression levels we generated a knockout of NOTCH3 in MCF7 cells using CRISPR/Cas9 and compared the NOTCH3 immunostaining with the parental MCF7 line. The results (Supplementary Fig. S5) showed virtually no background staining on knockout cells for anti-ECD (SF1559). The two anti-ICD antibodies D11B8 and ab23426 used in this study displayed some visible background staining in the nucleus, but not in the cytoplasm. Therefore, all three antibodies can be considered specific for endogenous levels of NOTCH3 staining in cytoplasmic puncta but unreliable for quantification of nuclear localisation.

**Fig. 5:**
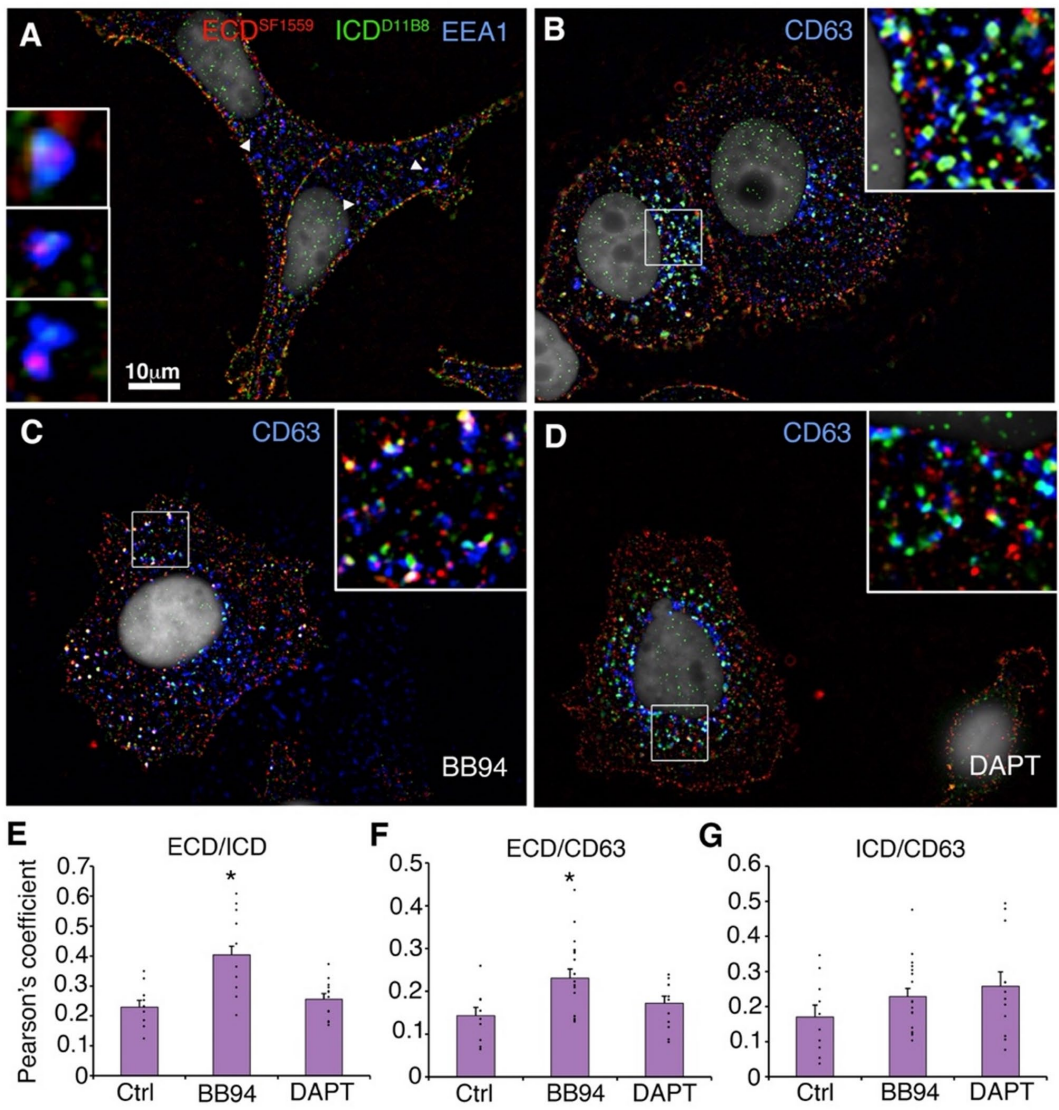
Effect of pathway inhibitors on endogenous NOTCH3 localisation in MCF7 cells. **(A)** EEA1-positive early endosomes of MCF7 cells costained with anti-ECD, and ICD. Arrowheads indicate endosomes shown enlarged in insets which have separate localisation of ECD and ICD puncta. **(B)** CD63-positive late endosomes costained with anti-ECD, and ICD. Boxed region is enlarged in inset showing late endosomes predominantly contain ICD-only puncta. **(C)** After treatment of MCF7 cells with metalloprotease inhibitor BB94, more full-length NOTCH3 staining is observed in the late endosomes, colocalised with CD63. **(D)** Treatment of cells with gamma-secretase inhibitor DAPT does not alter localisation of ECD in the late endosome. **(E-G)** Quantification of colocalization of ECD vs. ICD **(E)**, ECD vs. CD63 **(F)**, and ICD vs. CD63 (**G)**. Error bars, SEM, control DMSO-only treated cells *n* = 10, BB94 *n* = 18, DAPT *n* = 12. * indicates *p* < 0.05 by student t-test compared to control

Immunostaining of wild type MCF7 cells showed colocalisation of ECD and ICD around the perimeter of the cell, whereas NOTCH3 localisation within the cytoplasm could be as full-length, ECD-only or ICD-only labelled puncta. We detected all these forms in EEA1-labelled early endosomes (Fig. 5A), but ICD-only was more prominent in CD63 labelled late endosomes (Fig. 5B). When we treated the MCF7 cells with the metalloprotease inhibitor BB94 there was an increased presence of ECD colocalised with CD63 compared to control cells and full-length NOTCH3 could be visualised to be colocalised with CD63 (Fig. 5C, E,F). This is consistent with a role for ADAM10 in the shedding of ECD by S2 cleavage. In contrast, treatment of cells with the gamma-secretase inhibitor (DAPT) did not cause an increase in full-length NOTCH3 in the late endosomes (Fig. 5D-F) but there was a small observed increase in ICD localisation in the late endosome, consistent with the role of gamma-secretase in intramembrane S3 cleavage, which occurs after ECD shedding. However, this increase in ICD colocalisation was not statistically significant (Fig. 5G).

**Fig. 6:**
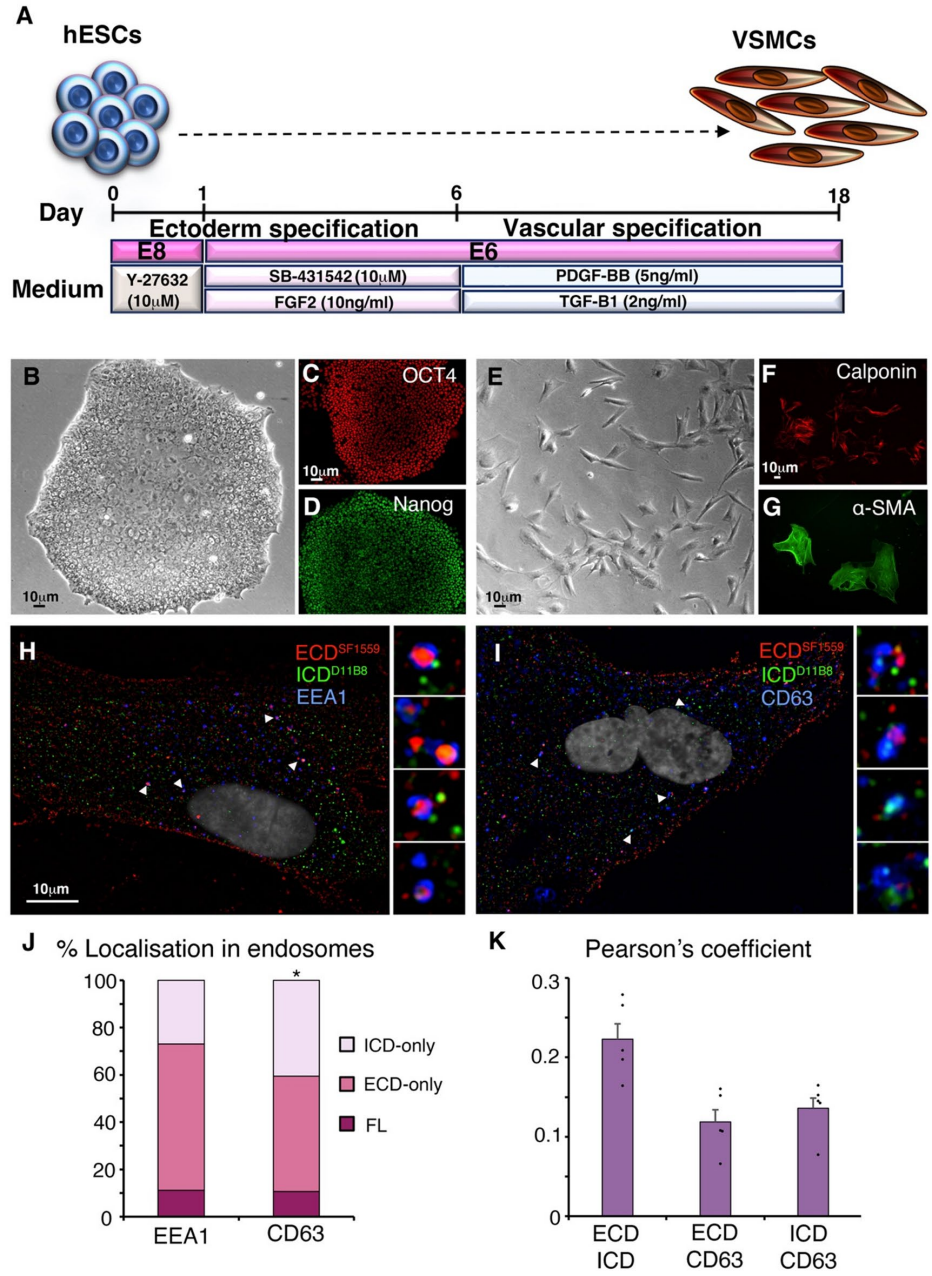
NOTCH3 ECD/ICD separation in endosomes of VSMCs derived from hESCs. **(A)** Summary of procedure followed to differentiate hESCs to VSMCs. **(B)** Brightfield image of undifferentiated hESC colony. **(C**,** D)** Undifferentiated hESC colony expressing stem cell markers OCT4 and Nanog. **(E)** Brightfield image of cells after differentiation protocol showing change of morphology. **(F**,** G)** After differentiation, cells express VSMC markers calponin and α-smooth muscle actin (SMA). **(H**,** I)** VSMCs immunostained to show separate localisations of ECD and ICD only puncta in early endosomes **(H)** and late endosomes **(I)**. Arrowheads point to endosomes shown enlarged in insets. **(J)** Quantification of % of NOTCH3 containing EEA1 and CD63 marked endosomes containing full-length, ECD-only and ICD-only puncta. * indicates *p* < 0.05, *n* = 674 NOTCH3-positive puncta from 2883 EEA1-positive endosomes scored and 1208 NOTCH3-positive puncta from 4550 CD63-positive late endosomes scored. Z-sections from 8 different cells were scored in each case. **(K)** Quantification of colocalisation between NOTCH3 and CD63 by Pearson’s coefficient shows similar relative distribution to expressed NOTCH3 in hTERT-RPE-1 cells (compare with Fig. 3I), *n* = 5 cells with mean of three z sections per cell scored, error bars are SEM

Since the CADASIL mutant phenotype is manifest in VSMCs, we differentiated human embryonic stem cells into VSMCs using established protocols [47, 48] (Fig. 6A-G), and confirmed differentiation by immunostaining for VSMC differentiation markers, Calponin and α-smooth muscle actin, and by change in morphology. We immunostained VSMCs for NOTCH3 ECD and ICD and costained for either EEA1 or CD63 (Fig. 6H-K). We found that there was separate localisation of ECD and ICD immunofluorescence within the cells. EEA1 positive endosomes had mostly ECD-only localised in the central lumen of the endosome, but full-length or ICD-only spots were also present (Fig. 6H, J). In CD63-positive late endosomes, puncta of ICD-only spots were present and increased in proportion compared to in EEA1 early endosomes (Fig. 6I, J). Colocalisation measured by Pearson’s coefficient (Fig. 6K) showed a similar relative distribution of NOTCH3 processed forms to expressed NOTCH3 in hTERT-RPE-1 cells (Fig. 3I). These results are therefore consistent with our observations on over expressed NOTCH3.

### Basal signal activation of WT and CADASIL mutant NOTCH3

**Fig. 7:**
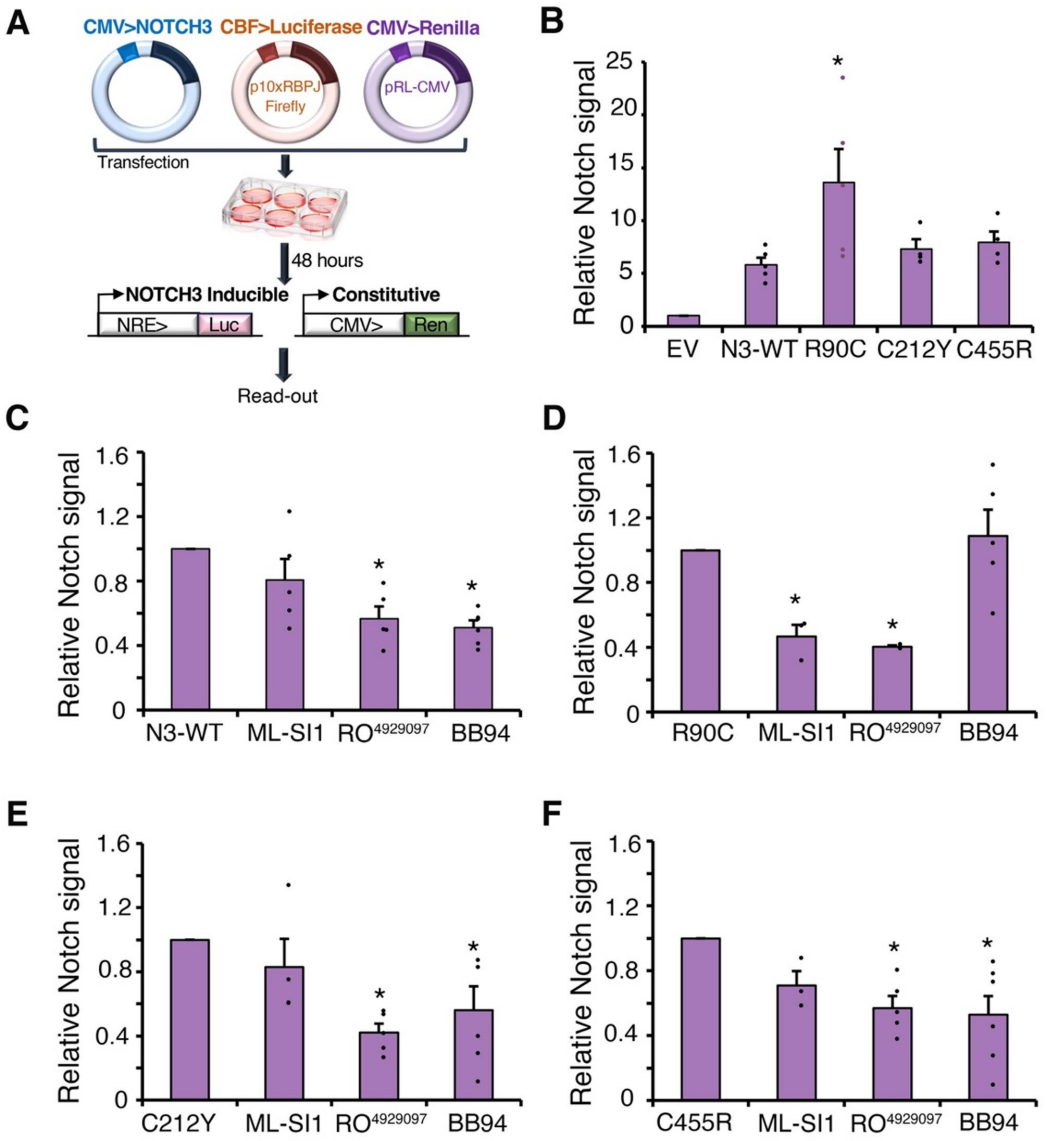
Basal NOTCH3 signalling requirements for WT and CADASIL mutants. **(A)** Schematic figure illustrating luciferase reporter assay methodology. **(B)** WT and CADASIL mutant NOTCH3 signalling after transfection into hTERT-RPE1 cells compared to empty vector (EV) control. * indicates *p* < 0.05 compared to WT NOTCH3 by student t-test. Error bars SEM, minimum of *n* = 4. **(C-F)** NOTCH3 signalling after treatment of transfected cells with TRPML inhibitor (ML-Sl1), gamma-secretase inhibitor (RO^4929097^), or metalloprotease inhibitor (BB94) for WT **(C)**, R90C **(D)**, C212Y **(E)**, and C455R **(F)** * indicates *p* < 0.05 compared with control by student t-test. Error bars are SEM, minimum of *n* = 3

We investigated whether NOTCH3 expressed in hTERT-RPE-1 cells was active in signalling. Cells were co-transfected with NOTCH3 expressing plasmid, a Notch response element (NRE) reporter driving luciferase expression, and a control plasmid that constitutively expresses Renilla for normalisation (Fig. 7A). Relative signalling increased after transfection with WT NOTCH3 and this was reversed by treatment of cells with gamma-secretase and metalloprotease inhibitors (Fig. 7B, C), but not by TRPML inhibitor. The latter affects the endolyosomal fusion/fission cycle [25, 49].

To investigate if CADASIL mutant NOTCH3 constructs are also active for signalling, equivalent amounts of the CADASIL mutant constructs were transfected into hTERT-RPE1 cells. A small increase in basal signalling observed for C455R and C212Y compared to WT was not significant, but R90C showed significantly increased activity (Fig. 7B). We next investigated whether signalling by CADASIL mutants involved the same or a different mechanism compared to WT NOTCH3 using the panel of inhibitors (Fig. 7C-F). Signalling by all constructs was reduced by gamma-secretase inhibitor. Unlike WT, R90C signalling was found to be metalloprotease-independent and but sensitive to TRPML inhibition (Fig. 7D). We found that C455R and C212Y were sensitive to metalloprotease inhibition and were less sensitive to TRMPL inhibition, hence these constructs signalled more like WT (Fig. 7E, F). Thus, the increased signal activation of R90C mutation was associated with a switch in activation mechanism. A small decrease in signalling after TRPML inhibition that was observed for C455R which, although not significant (*p* = 0.08), may nevertheless hint that the selection of activation mechanism is not all or nothing and that both mechanisms may contribute to overall levels of activity for some mutants.

Our results therefore demonstrate both heterogeneity of the cellular location where ECD shedding occurs and distinct basal activation mechanisms of different CADASIL mutants.

## Discussion

We investigated the endocytic trafficking and processing of human NOTCH3 and compared WT and three CADASIL mutant variants. We found that, contrary to expectations, the secretory pathway trafficking of expressed mutant NOTCH3 was not blocked in the ER and both WT and mutant NOTCH3 were transported to the cell surface and displayed a similar punctate distribution. This indicates that protein misfolding is not contributing to CADASIL mutant outcomes in the cell context studied here. Modelling of mutant domains showed that amino acid changes could be incorporated without gross alterations to the EGF-fold. While we did not observe differences in the way WT and mutant NOTCH3 are presented at the cell surface, we do not preclude more subtle quantitative differences in the S1 processing and transport of NOTCH3 to the cell surface. R90C for example has a small but significant increase in accumulation of NOTCH3 in the ER and upshifted forms observed on Western blots can be inferred to be not S1 processed. How this biochemical difference impacts on further processing, trafficking and signalling of R90C is not clear. Transient transfection is a limitation of our study and further work with endogenous mutants or clonal stable cell lines will be needed to elucidate these biochemical differences between different mutants. For example, it has been reported that a stable cell line expressing mouse NOTCH3^R142C^ had reduced S1 processing and reduced presentation of the mutant NOTCH3 at the cell surface compared to WT [43]. Nevertheless, once at the cell surface, following pulse chase endocytic uptake experiments, we showed that WT and all mutant NOTCH3 constructs were endocytosed similarly, becoming localised to the early endosome after 15–30 min of the chase period. Interestingly, we found both full-length (colocalised ECD and ICD staining) and ECD-only spots in the early endosomes, the latter normally located in the endosomal lumen. This indicates that, following uptake of full-length NOTCH3, ECD shedding occurs in the endocytic pathway. Even after a one-hour chase we found little surface-labelled ECD in the late endosomes, although ICD was present. We could not distinguish whether this was due to ECD removal in the early endosome or because the endocytosed full-length NOTCH3 had not yet reached this location. However, when total NOTCH3 was immunostained in fixed/permeabilised cells, we also found a predominance of ICD-only staining in the late endosome and lysosome compared with ECD. The punctate distribution of NOTCH3 within these structures is consistent with previous observations in *Drosophila* cells in which Notch was found to occupy distinct subdomains of the endosomal membranes [26]. It is interesting to speculate whether S1 cleavage by Furin is needed to allow dissociation of ECD and ICD within the endosomal pathway. However, because of the requirement for S1 cleavage for trafficking of NOTCH3 to the membrane it is difficult to separate these requirements. For ligand-activated signal, even though the S1 cleaved heterodimer is non-covalently associated it still requires S2 cleavage by ADAM10 to release the ECD. However, during endocytosis perhaps pH related changes could make the S1 heterodimer more prone to separation. Nevertheless, basal signalling by WT, C212Y and C455R mutant NOTCH3 was still sensitive to metalloprotease inhibitors, although there remains the possibility that R90C dissociation could be Furin-dependent. Further work is needed to investigate this possibility.

There are several reasons that immunofluorescence images could produce an apparent separate localisation of ECD and ICD epitopes even if NOTCH3 was present as full-length protein. We considered the possibilities of non-specific staining of either ECD or ICD antibodies, epitope masking, and a N or C-terminal truncation which results in near full-length NOTCH3 that lacks one of the terminal epitopes. All antibodies used in this study were specific to NOTCH3 expressing cells and not detectable in non-transfected cells at the exposures used, so all the staining observed could attributed to NOTCH3. To rule out epitope masking we used different antibody combinations to double label either the ECD or the ICD with differently located epitopes. We found ICD-only spots that lacked both ECD epitopes and we found ECD-only spots lacking both ICD epitopes. This supports the conclusion that true separation of ECD and ICD was the explanation for our imaging data since it is unlikely that both epitopes of the ECD or the ICD were masked at the same time. Only one epitope recognised by the rat monoclonal 8G5 antibody showed signs of epitope masking. While 8G5 stained the lysosome and nucleus of NOTCH3 expressing cells, it was barely detected in the early endosome or the cell membrane. This epitope exclusion was not observed for other anti-ICD antibodies used in this study. To further test our conclusions, we used double FP-tagged NOTCH3 as an alternative means to observe ECD/ICD separation that is not reliant on immunostaining and obtained results that support the finding that NOTCH3 is full-length at the cell membrane with ECD separation occurring in the endosome. The location of the ECD tag between the most C-terminal EGF modules and the NRR also implies that the majority of the ECD is removed together rather than N-terminal fragmentation being the cause of epitope separation. The eventual fate of the ECD remains uncertain. Since ECD will be oriented to the luminal side of the endosome membrane then following its separation from ICD it would be expected to be transferred to intraluminal vesicles, this may lead to further processing and ultimately its degradation on fusion with lysosomes. Alternatively, ECD may enter recycling endosomes to be secreted, or be expelled from the cell within exosomes, and both are possible routes to its deposition in GOM.

We investigated the possibility that the observed endocytic processing pathway was a result of over expressing NOTCH3. Two cell types were used, MCF7 which is a breast cancer cell line that expresses NOTCH3, and VSMCs differentiated from hESCs. The specificities of the antibodies used at these levels of endogenous expression were confirmed using a NOTCH3-knockout MCF7 cell line, generated by CRISPR/Cas9 induced non-homologous end joining. In both MCF7 cells and VSMCs we saw similar independent ECD and ICD epitope localisation patterns as seen for over expressed cDNA in hTERT-RPE1 cells. Furthermore, when we treated MCF7 cells with the metalloprotease inhibitor BB94 we saw increased presence of ECD in the late endosome, colocalised with ICD and presumably representing increased trafficking of NOTCH3 full-length protein to the late endosome. This supports an intracellular location for ECD removal and demonstrates that the reduced ECD presence in the late endosome in untreated cells is not due to poor penetrance of the ECD antibodies into the late endosomal lumen. A limitation of this work is that it was performed in hTERT-RPE-1 cells which are unrelated to the CADASIL condition. We used these cells as a generic cell line with a track record of use in protein trafficking studies which had a low background of endogenous NOTCH3. However, Notch proteins function similarly in a wide variety of developmental contexts and in many different cell types. We expect therefore that our findings will be widely applicable across cell types, including in VSMCs. This expectation is supported by similar findings on endogenous NOTCH3 localisations in both MCF7 cells and VSMCs. More detailed biochemical analysis of mechanisms will require future work in stable cells lines or cells endogenously expressing mutant NOTCH3 genes.

To characterise the signalling activity for WT and CADASIL mutants we used a reporter assay that expresses luciferase in response to Notch pathway activation and treated cells with different pathway inhibitors. We found that expressed full-length NOTCH3 had basal activity that was dependent on metalloprotease and gamma-secretase activity. R90C showed significantly increased signalling activity compared to WT and shifted to an alternative mechanism of activation that was independent of metalloprotease activity and dependent on TRPML, a calcium channel protein involved in endosomal/lysosomal fusion and fission. TRPML is known to be involved in ADAM10-independent endosomal Notch activation pathways in *Drosophila* [25]. An observed increase in signalling activity of C455R and C212Y mutants was not statistically significant compared to WT, and unlike R90C the activity was reduced by the metalloprotease and gamma-secretase inhibitors. Signalling by WT, C455R and C212Y were less affected by TRPML inhibition than R90C, again pointing to distinct activation mechanisms for basal signalling between R90C and the other constructs. This shift in mechanistic requirements was correlated with an altered endocytic localisation, with ECD removal likely enhanced in the EEA1 early endosome or occurring before reaching this location. The signalling activity of the C455R mutation is informative as it strongly reduces ligand-dependent induction of NOTCH3 signalling [22, 24]. It is likely therefore that the basal signalling we observe is in large part due to a similar trans ligand-independent activation mechanism as has been observed for *Drosophila* Notch [25], which can arise from either ADAM10-dependent or independent mechanisms. This is consistent with previous work indicating that full-length wild type NOTCH3 has a basal level of constitutive activation [27, 28]. We do not rule the involvement of cis-expressed ligands in the regulation of NOTCH3 endocytic trafficking and activation [50], but it is noteworthy that the cis-interaction binding site of canonical Notch ligands is thought to occur through the same binding region as the trans-ligand interaction and is therefore also likely to be disrupted by the C455R mutation [51].

The increased activity of CADASIL mutants, particularly R90C, is intriguing and is consistent with a number of recent reports which have suggested that CADASIL reflects a gain of function NOTCH3 activity. Increased NOTCH3 signalling has previously been identified in VSMC primary cultures derived from CADASIL patients [16]. CADASIL VSMCs were found to have increased cell proliferation, increased apoptosis and altered cytoskeletal morphology. NOTCH3 activation in the transgenic NOTCH3^R169C^ mouse CADASIL model was also increased [52]. Increased activation of NOTCH3 in CADASIL was additionally observed in studies of VSMCs differentiated from induced pluripotent stem cells (iPSCs) generated from the fibroblasts of a CADASIL patient [53]. In another study, CADASIL mutant VSMCs, also differentiated from patient-derived iPSCs, were found to be more prone to apoptosis and failed to stabilise endothelial networks in a co-culture model, unlike wild-type VSMCs [48]. These defects were reversed by siRNA knockdown of NOTCH3, suggestive of a gain-of-function effect. Interestingly, in *Drosophila*, a number of Cysteine substitution mutants in EGF domains have been identified that display dominant, gain of function phenotypes [54]. Our work now offers a mechanistic explanation for increased Notch activity derived from EGF-module mutants that result in free cysteine mutants in Notch, through a ligand-independent activation pathway, involving shedding of the ECD during endocytic trafficking. We further suggest that subtle increases in this process over the long term may contribute to observed accumulation of ECD in CADASIL patients. The finding that R90C shifts its activation mechanism to a metalloprotease-independent mode indicates there is underlying heterogeneity in the ways the ECD can be detached from the full-length protein. This insight may offer routes to develop targeted therapies linked to specific mechanisms associated with particular mutants. Since R90C is amongst the most frequently occurring CADASIL mutants [36] then identifying a targeted treatment for this mechanism would represent a significant step forward in the treatment of inherited vascular dementia. Future work could identify components needed only for the R90C activation route and our findings could be tested in vivo through knockout or knockdown of these components in mouse CADASIL models expressing the R90C construct. It remains to be seen whether other CADASIL mutants also switch to the alternative activation route. Even the mutants used in this study, which here activated like wild type, may switch to alternative mechanisms as the disease progresses or in response to additional environmental factors or other modifiers. For example, in *Drosophila*, switching between alternate metalloprotease-dependent and independent mechanisms can be brought about by changes in expression of ubiquitin ligase regulators that modify endosomal trafficking destinations of Notch [26]. Downregulating the function of ESCRTIII complex proteins, which regulate transfer of proteins between the endosomal membrane and the lumen, also activates both *Drosophila* Notch and WT human NOTCH3 in a metalloprotease-independent manner [26].

There have been few studies that have addressed how heterogeneity of patient outcomes might be linked to differently located mutants. Recent data has suggested that CADASIL mutants in the N-terminal region have earlier onset of disease symptoms than more distally located mutants [55]. Other studies involving genome sequencing of large cohorts of individuals have revealed that CADASIL-like mutations in NOTCH3 are more common in the asymptomatic population than has been previously thought [11]. Such mutations have been shown to be predisposing to spontaneous stroke [11]. Altered NOTCH3 activity has also been linked to age-dependent small vessel disease [6] which, like CADASIL is linked to impaired VSMCs and is an important contributor to stroke and vascular dementia, the latter comprising around 20% of all dementia diagnoses [1, 56]. Around 50% of people aged over 65 have signs of SVD on autopsy [18]. Finding ways to tune NOTCH3 levels through modulation of its endocytic regulation may provide route to therapies for both inherited and age-dependent forms of these conditions.

## Electronic supplementary material

Below is the link to the electronic supplementary material.

Supplementary Material 1

## Data Availability

No datasets were generated or analysed during the current study.
