# Supplementary material for: Extracellular domain shedding of NOTCH3 during endocytosis associated with heterogeneity between different CADASIL mutant activation mechanisms
Source: Cell Commun Signal. 2025 Aug 6;23:366. doi: 10.1186/s12964-025-02362-1 (PMC12326875; doi:10.1186/s12964-025-02362-1)
Supplement: Supplementary file 1 — Supplementary Material 1 [file 12964_2025_2362_MOESM1_ESM.pdf]

Supplementary Figures

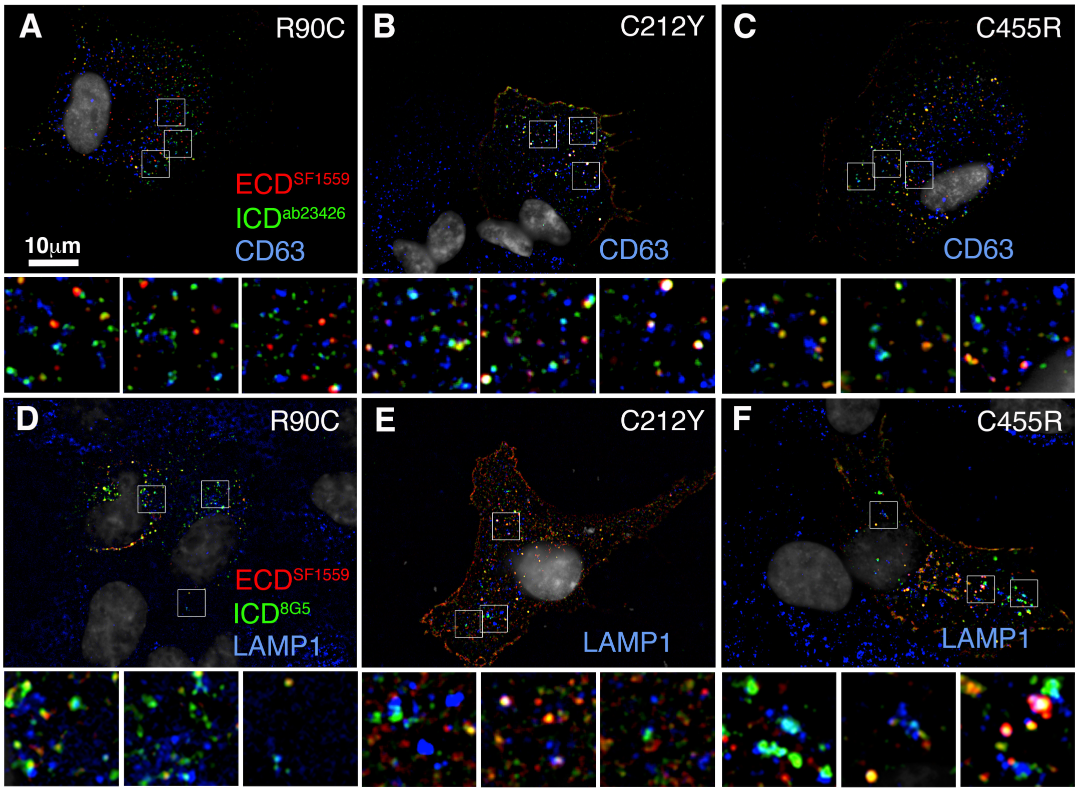

Fig. S1. CADASIL mutant localisation in late endosomes and lysosomes. (**A-C)** Localisation of ECD and ICD compared to CD63-positive late endosomes, for R90C **(A)**, C212Y **(B)** and C455R **(C)**. Separated ICD puncta can be observed in late endosomes for all three mutants. **(D-F)** Similarly ICD-only puncta can be observed in LAMP1-positive lysosomes for R90C **(D)**, C212Y **(E)** and C455R **(F)**.

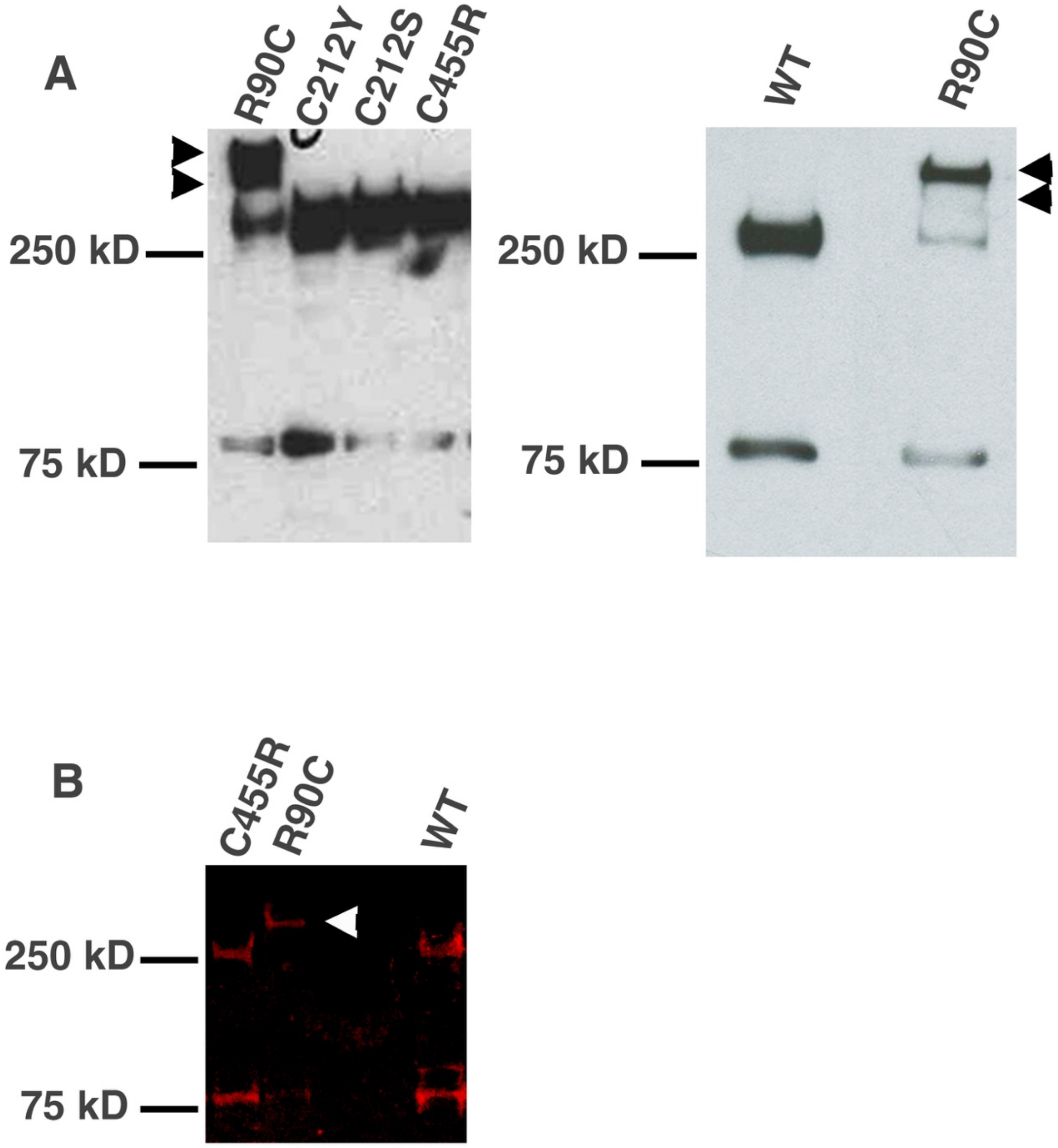

Fig. S2. Western blots of expressed WT and CADASIL mutant NOTCH3. **A)** Cell lysates of WT and mutant NOTCH3 expressing HEK-293 cells were western blotted with anti-NOTCH3 ICD (ab-23426). Arrow heads indicate additional bands detected for R90C which migrate at higher molecular weight than full length WT protein, which were not detected for the other mutants. **B)** Cell lysates of WT and mutant NOTCH3 expressing hTERT-RPE1 cells, western blotted with anti-NOTCH3 ICD (ab-23426), white arrowhead indicates upshifted R90C band.

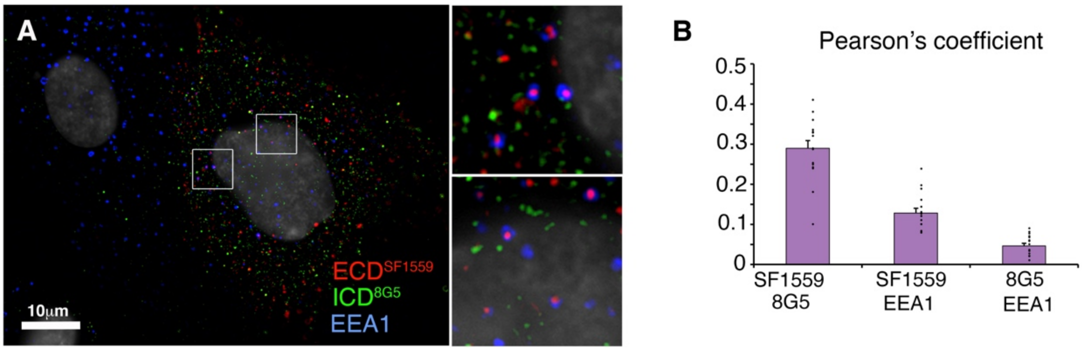

Fig. S3. Anti-ICD 8G5 epitope staining is excluded from early endosome location. **(A)** NOTCH3 expressing cell immunostained to compare the localisation of ECD and ICD-8G5 in EEA1-marked endosomes. ECD is clearly present in EEA1-endosomes, but there is little ICD-8G5 staining present in the endosome. Boxes show the regions enlarged in insets. **(B)** Quantification of the epitope colocalisations that are illustrated in A. Error bars are SEM, n=16 cells.

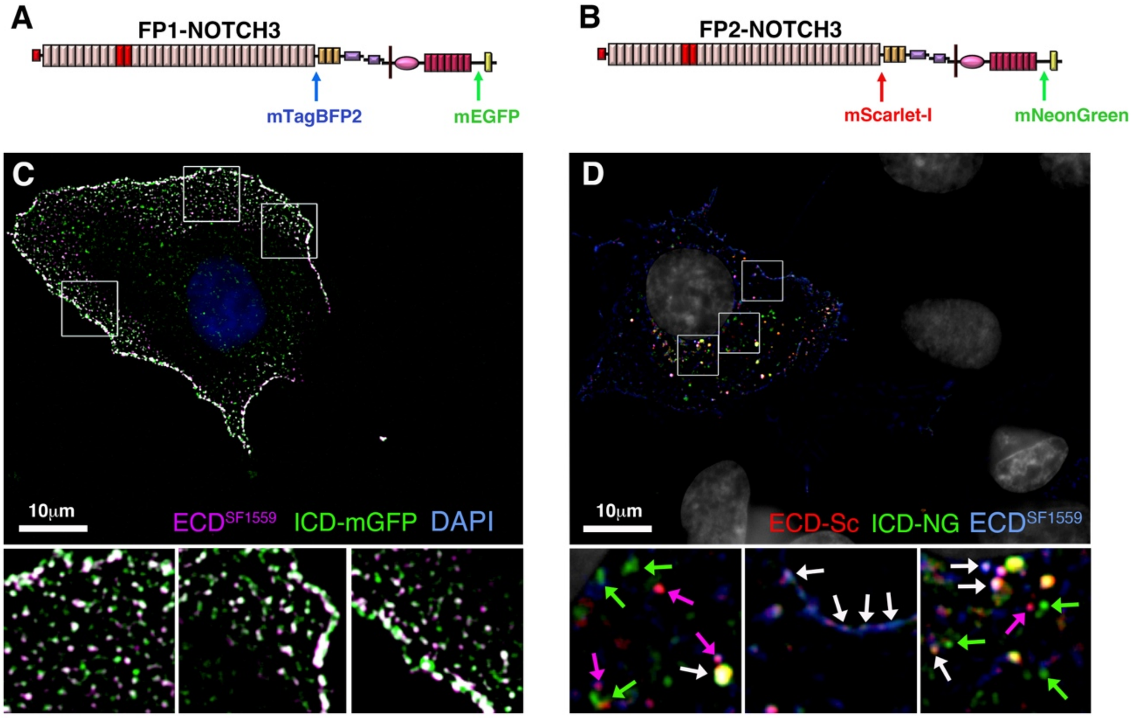

Fig. S4. Fluorescent-tagged NOTCH3 shows ECD/ICD separation in intracellular compartments. **(A,B)** Schematic figure showing insertion sites of fluorescent-tagged proteins in WT NOTCH3 expression constructs FP1 and FP2. **(C)** Immunostaining of non-permeablised cells of surface-localised FP1-NOTCH3 with anti-ECD, which shows colocalisation in surface puncta with ICD-GFP, showing that full-length NOTCH3 is localised to the cell membrane. **(D)** Immunostaining of permeablised cells of FP2-

NOTCH3. As well as full-length FP2-NOTCH3 (white arrows), separated mScarlet-I puncta colocalises with anti-ECD staining (magenta arrows) and ICD-GFP puncta can be seen which lack either mScarlet-I or anti-ECD staining (green arrows).

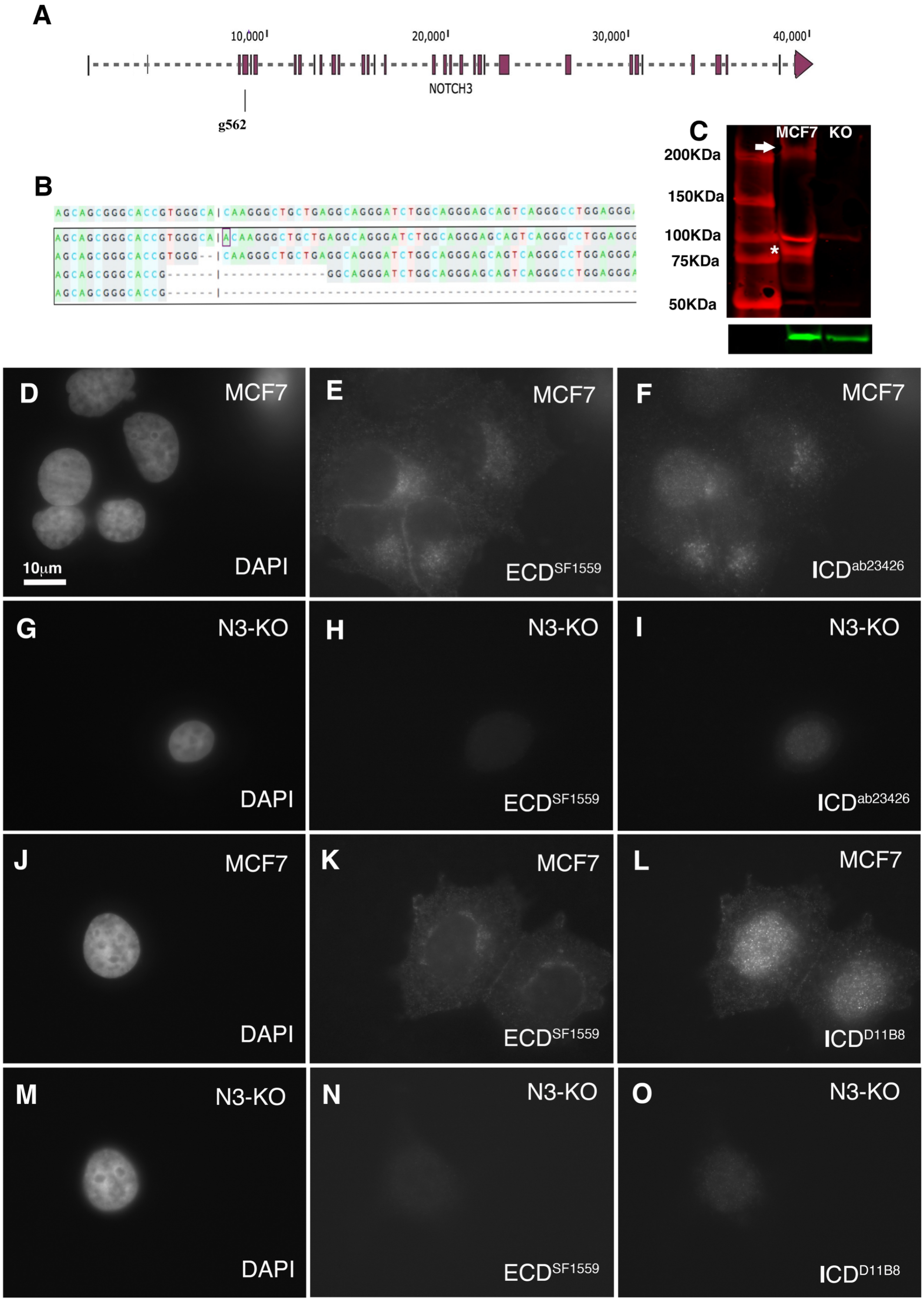

Fig. S5. Validation of antibodies for immunolocalisation of endogenous NOTCH3. **(A)** NOTCH3 gene structure. Exons depicted by pink bars. The location of the guide RNA (g562), used for generating the CRISPR/Cas9 NOTCH3 knock out (N3-KO) in MCF7 cells is indicated. **(B)** After gene editing,

sequencing of NOTCH3 genomic DNA, from line 2C7, in vicinity of guide site, revealed four sequences (boxed) containing deletions with frame shifts, and no WT sequence was identified. **(C)** Western blot stained with anti-ICD^{D11B8} (red) showing loss of NOTCH3 expression in N3-KO cell line. Arrow indicates full-length NOTCH3 and asterisk indicates membrane-tethered ICD. A band around 100kDa is likely a non-specific binding. **(D-O)** Immunofluoresence staining of fixed and permeabilised parental MCF7 cells **(D-F, J-L)** and the 2C7 NOTCH3 knock-out line **(G-I, M-O)** for DAPI **(D, G, J, M)**, anti-ECD^{SF1559} **(E, H, K, N)**, anti-ICD^{ab23426} **(F, I)** and anti-ICD^{D11B8} **(L, O)**. Antibody staining of parental MCF7 cells is distinctly above minimal background staining of ECD^{SF1559} in NOTCH3-KO. While both ICD antibodies show some immunostaining of the nucleus in the NOTCH3-KO line, there is minimal staining in the cytoplasm compared to stronger staining in parental cells.
